# Seven perspectives on GPCR H/D-exchange proteomics methods

**DOI:** 10.12688/f1000research.10667.1

**Published:** 2017-01-30

**Authors:** Xi Zhang

**Affiliations:** 1 Independent Researcher, Montreal, QC, H2Y 1H3, Canada

**Keywords:** GPCR, H/D-exchange, lipids, membrane proteins, detergents, structural proteomics

## Abstract

Recent research shows surging interest to visualize human G protein-coupled receptor (GPCR) dynamic structures using the bottom-up H/D-exchange (HDX) proteomics technology. This opinion article clarifies critical technical nuances and logical thinking behind the GPCR HDX proteomics method, to help scientists overcome cross-discipline pitfalls, and understand and reproduce the protocol at high quality. The 2010 89% HDX structural coverage of GPCR was achieved with both structural and analytical rigor. This article emphasizes systematically considering membrane protein structure stability and compatibility with chromatography and mass spectrometry (MS) throughout the pipeline, including the effects of metal ions, zero-detergent shock, and freeze-thaws on HDX result rigor. This article proposes to view bottom-up HDX as two steps to guide choices of detergent buffers and chromatography settings: (I) protein HDX labeling in native buffers, and (II) peptide-centric analysis of HDX labels, which applies (a) bottom-up MS/MS to construct peptide matrix and (b) HDX MS to locate and quantify H/D labels. The detergent-low-TCEP digestion method demystified the challenge of HDX-grade GPCR digestion. GPCR HDX proteomics is a structural approach, thus its choice of experimental conditions should let structure lead and digestion follow, not the opposite.

## Abbreviations

**GPCR**, G protein-coupled receptor; **HDX**, H/D-exchange; **TM**, transmembrane; **DDM**, n-dodecyl-β-D-maltopyranoside; **TCEP**, Tris-2-carboxyethylphosphine; **DLT**, DDM-low-TCEP; **CHS**, cholesteryl hemisuccinate; **β _2_AR**, β _2_ adrenergic receptor; **C** ***c*** **O**, cytochrome *c* oxidase; **TSPO**, translocator protein; **UPLC**, ultra-performance LC; **EM**, electron microscopy; **LCP**, lipidic cubic phase; **PC**, phosphatidylcholine; **DPC**, dodecyl phosphatidylcholine (12:0); **DMPC**, dimyristoyl phosphatidylcholine, 1,2-dimyristoyl- *sn*-glycero-3-phosphocholine (14:0/14:0); **PO**, 16:0/18:1, 1-palmitoyl-2-oleoyl; **DO**, 18:1/18:1, 1,2-dioleoyl; **PE**, phosphatidylethanolamine; **PG**, phosphatidylglycerol; **PS**, phosphatidylserine; **PA**, phosphatidic acid; **PI**, phosphatidylinositol; **PIPn**, PI phosphate.

## Introduction

This opinion article is a response to the recent call to “strive for reproducible science” ^1^. January 2010 saw the publication of the first fully automated membrane protein bottom-up H/D-exchange (HDX) proteomics method, which can map human G protein-coupled receptor (GPCR) dynamic conformations in solution at repeated HDX coverage of 89%, out of ~90% MS/MS coverage ^2^. This method broke the years-long sub-25% coverage impasse, provided the first useful HDX proteomics protocol to obtain meaningful structural information of seven-transmembrane (TM) GPCR for drug discovery, and established HDX proteomics as a powerful mainstage tool for GPCR structure-function investigation. These peptides were robustly reproduced in over two hundred independent HDX runs, using several ligand-states of prototypic human GPCR β _2_ adrenergic receptor (β _2_AR) from numerous batches of purifications ( 2 and unpublished study by Xi Zhang and Patrick R. Griffin, *et al.*). Enabled by a DDM-low-TCEP (DLT) digestion method (DDM, n-dodecyl-β-D-maltopyranoside; TCEP, Tris-2-carboxyethylphosphine), this protocol integrates autosampler control programs to coordinate continuous full sets of HDX incubation, online digestion and data acquisition of high-performance liquid chromatography mass spectrometry (HPLC MS), and is flexible for users to choose 0-to-3600-second or longer-hour incubation modules, and regular or longer HPLC for MS/MS sequencing. Subsequently, this protocol has been applied in large-scale GPCR efforts and attracted broad interests from the GPCR community ^3– 9^.

However, mis-representations have also emerged ^3, 6^, reflecting misunderstanding of the GPCR HDX proteomics approach at multiple levels. Outstanding problems include: confusing the HPLC MS/MS and HPLC MS steps; confusing the various roles of detergents; incorrectly claiming the 2010 study analyzed HDX-labeled peptides with 120-min HPLC MS experiment; and calling the 89% HDX coverage invalid ^3^. Questionable procedures include: neglecting the HPLC MS/MS part of HDX and the critical optimization of pepsin column digestion ^3^; destabilizing membrane proteins by dilution with zero-detergent buffers and introducing Na/K interference to the GPCR protein system, such as using a quench/digestion buffer composed of 20 mM TCEP and 0.1 M KH _2_PO _4_ pH 2.01 to dilute GPCR DDM/NaCl solution ^3^; disturbing proteins or labels with extra freeze-thaws ^3^; neglecting the effects of the bicelle detergents, lipids and adducts on MS, data-dependent MS/MS acquisition and peptide identification ^3^; and switching to manual mode for membrane protein HDX. Backed by these problematic procedures, the JASMS May 2015 paper repeatedly claimed CHAPSO/DMPC (dimyristoyl phosphatidylcholine, 14:0/14:0) bicelle specifically as a “better solubilization method than DDM for HDX-MS analysis of GPCRs” ^3^, but minimized discussing the structural concerns of CHAPSO/DMPC on proteins. The peptide MS spectra of CHAPSO/DMPC appeared unusually noisy compared with DDM ^3^, raising questions about potential effects on spectrum quality and HPLC column health over long-term practice. Meanwhile, another study submitted in May 2015 reported that human β _2_AR purified from Sf9 did not predominantly sequester PC nor 14:0/14:0 chains from membrane, but enriched for cholesterol by 17.7 fold, and 18:1/18:1 chains by 80 fold ^10^, raising structural concerns against CHAPSO/DMPC.

This opinion article reasons that these problems reflect a common lack of systematic thinking and confusions of fundamentals in the GPCR bottom-up HDX proteomics approach, such as the MS *versus* MS/MS step, the protein structure HDX labeling *versus* label analysis step, and the roles of detergent/lipid additive for structure *versus* for digestion. Given the recent surging interest to study GPCRs using bottom-up HDX ^3– 9, 11, 12^, this viewpoint clarifies critical technical nuances and logical thinking, and emphasizes systematically considering membrane protein structure stability and HPLC MS compatibility, throughout the pipeline. This article explains bottom-up HDX as two flexibly coupled modules to guide choices of detergent buffers and HPLC settings, and highlights the effects of metal ions, zero-detergent shock and freeze-thaws on membrane protein structure, stability and HDX result rigor. Rather than a comprehensive overview of HDX or membrane protein methods ^8, 9, 12, 13^, or a refutation of particular publications, this article aims to provide a systematic practical guide to help scientists overcome cross-discipline pitfalls, and understand and reproduce the GPCR HDX methods at high quality. The ignorance of these nuances, rather than the lack of care or diligence, likely caused the previous impasse and emerging problems and endangers future success. Strengthened by important non-HDX biophysics studies published after 2010, these seven first-hand insights are critical to clear emerging misconceptions, but are not discussed in the original 2010 report.

## 1. Deep-sequencing-based bottom-up HDX MS: a two-stage analysis

**Figure 1.**
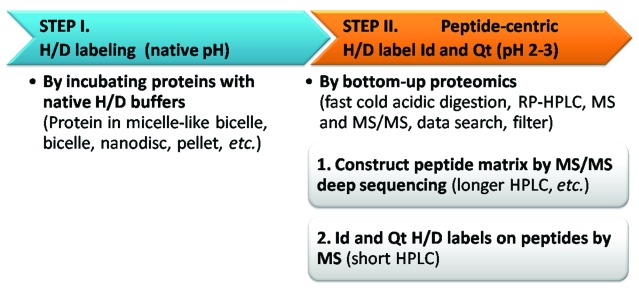
Two-step experiments of the deep-sequencing-based bottom-up differential HDX proteomics method for GPCR. Id, identification; Qt, quantitation.

Although HDX descriptions usually list multiple steps and elaborate on the well-established logistics of bottom-up proteomics ^3, 6^, what has critically enabled the membrane protein HDX breakthroughs ^2, 14^ is to think in terms of two distinct yet flexibly-coupled modules beyond the routines ( Figure 1). The overall method workflow of bottom-up HDX structural proteomics of membrane protein GPCR can be viewed as two steps: (I) label via H/D-exchange, and (II) analyze—identify and quantify—H/D labels using bottom-up proteomics. H/D label analysis is peptide-centric and also has two stages: (1) identify and construct peptide matrix (a set of reproducibly identified peptides) using HPLC MS/MS deep sequencing, and (2) quantify H/D-labels in MS for each identified peptide, using MS peak area summed from the peptide’s isotopic envelope ( Figure 1). These two stages share the same protease digestion method and as similar as possible temperature and HPLC-MS instrument, but can differ in some other HPLC and MS/MS or MS conditions to best fulfill distinct purposes.

The MS/MS deep sequencing stage aims to identify as many robust peptides as possible for target proteins. TM sequences often fall short of ionization efficiency (overdigestion is explained below), thus a longer HPLC gradient is desired to simplify elution population and allow these peptides a better chance to get picked for MS/MS scan in the ion-abundance-ranked data-dependent acquisition. Because these peptides do not carry H/D-labels, longer HPLC MS/MS analysis time causes no harm here. To obtain a robust MS/MS peptide matrix, the 2010 protocol then matched and iteratively filtered these MS/MS spectra using a multilayer method: (1) each peptide should score above 20 in MASCOT search against the target sequence, but spectra stay unmatched in decoy search against the reversed sequence (most decoy matches scored way below 10); (2) peptide sequences should comply with the pepsin preference sites reported by Hamuro *et al.* in 2008 ^15^; (3) fragment ions in MS/MS spectra appear reasonable in manual inspection; and (4) precursor ions should be repeatedly confirmed in high-resolution MS using the HDX MS experiment’s HPLC gradient. This MS/MS stage provides an initial peptide matrix, which is further refined in subsequent HDX MS.

However, restricted by the minutes’ time window of HDX pipeline to minimize H/D label back-exchange, the HDX MS stage uses shorter HPLC gradient and just MS scans. Peptide identification in HDX MS data is based on: (1) accurate peptide mass matching to those in the pre-constructed MS/MS peptide matrix; (2) retention time reproducibility over all HDX runs and correlation with the longer gradient; and (3) iterative confirmations via checking consistency across redundant peptide ladders, multiple charge states, and overall HDX profile trend throughout the H/D-incubation time points. Targeted MS/MS may further confirm ambiguous peptide ions. Ideally peptide MS/MS identification should be performed at the same HPLC gradient as used in HDX MS quantitation, but it challenges the capacity and scan speed of current popular HPLC and mass spectrometer instruments, and proved often unnecessary for simpler purified protein samples on high-resolution orbitrap analyzers ( 2, 16 and unpublished study by Xi Zhang and Patrick R. Griffin *et al.*). Nonetheless, the rapidly growing data-independent acquisition MS/MS, which uses wide precursor isolation window for simultaneous fragmentation, may reconcile this gap ^17– 20^.

Therefore, constructing the MS/MS peptide matrix favors longer HPLC gradient for exhaustive identification (no H/D labels), but the MS-based H/D label quantitation can apply short HPLC to minimize H/D label loss, as this step is based on MS peptide mass matching. The 2010 protocol achieved the 89% HDX coverage by devising a total 9.5-min HPLC method for HDX MS ^2^, not the 120 min claimed by *Duc et al.* ^3^. This short HPLC for HDX MS was repeated in subsequent large-scale GPCR HDX studies. As a part of the 2010 strategy, changing from the regular 60-min to the 120-min HPLC method for MS/MS sequencing successfully recovered multiple TM peptides, and they were robustly identified throughout HDX MS mapping.

## 2. DDM as a tool for making structural-grade protein versus a tool for digestion

DDM/cholesteryl hemisuccinate (DDM/CHS) bicelle-like micelle served as a tool to prepare upstream structural-grade membrane protein solution samples, and to mark these conformations with matching D _2_O buffer. As a tool for downstream digestion, DDM-low-TCEP alone suffices to support protease activity and to solubilize and stabilize substrates against aggregation throughout digestion. Importantly, the combination of these two modules—protein preparation-labeling and digestion—is flexible ( Figure 1).

Upstream protein states can vary vastly with sample preparation methods, which should thus be screened with rigorous function assays (multi-facet, including activity, ligand binding and stability) and matched by the H/D-labeling buffer. However, this does not void the broad utility of DLT method for downstream digestion. Not only can DLT digestion be applied to various upstream protein preparations, including myriad detergent/lipid bicelles, lipid bilayer nanodiscs, DDM/CHS bicelle-like micelle, membrane pellets and intact organelles ^21^, but also DLT HDX-proteomics provides a tool to visualize their different effects on protein in-solution conformations. Remarkably, the DLT digestion method proved highly compatible with soluble protein projects to share the same regular reversed-phase (RP)-HPLC ESI MS and MS/MS instrument platform. Across large-scale applications, no deterioration was observed in peptide MS spectra (smooth not noisy), column health or sample carryover, similar to non-DLT soluble proteins ( 2 and unpublished data by Xi Zhang and Patrick R. Griffin, *et al.*) ( Supplementary Figure 1 and Supplementary Figure 2B). Therefore, the HDX-grade digestion of GPCRs is technically solved and is no longer hampered by solubilization during the digestion.

By contrast, CHAPSO/DMPC is less suitable as a tool for digestion in broader proteomic applications. Both CHAPSO and DMPC form net strong fixed positive charges under acidic pH, combined with high concentration (CHAPSO critical micelle concentration cmc is 8 mM, 5x cmc is 40 mM), they are long observed to dominate ionization, likely interfere with peptide RP-HPLC data-dependent MS/MS, and may harm long-term RP-HPLC column health, despite possible chromatograph improvement in ultra-performance LC (UPLC). Even anionic cholate entailed UPLC ^22^, and anionic deoxycholate (cmc 6 mM, no fixed positive charges) proved to require removal by ethyl acetate extraction before HPLC injection ^23^. Samples that contain CHAPS, similar to CHAPSO (same charged groups, same 8 mM cmc, one less hydroxyl) find routine rejections at proteomics facilities: “Non acceptable buffers include NP40, CHAPS, Triton X, and PEG” ( https://mass-spec.stanford.edu/sample-preparation; Sample Preparation, Stanford University Vincent Coates Foundation Mass Spectrometry Laboratory; Sept 28, 2015 access).

## 3. CHAPSO/DMPC bicelle versus DDM/CHS bicelle-like micelle: not unique to the HDX approach

How the presentation methods influence membrane proteins’ native structures is the premier concern common to all solution-based biophysical approaches that aim to measure their functional/native states ^14, 24^. For solution-based structural technologies, the freedom from high-resolution crystallogenesis—itself a quality control of how comfortable (though not always native) membrane proteins are in these conditions—presents both advantages and pitfalls, and calls for extra rigor and caution in protein handling, data interpretation, and cross-examination with other function and structure measurements. To avoid masking the effects of intended perturbations, such as ligand stimulation, protein buffers often aim to approach native-like and function-neutral: stabilize the protein and minimize distortion (deactivation or over-activation). 

The 2010 study prepared human GPCR protein in DDM/CHS solution ^2^, because mammalian GPCR natural habitats include 20–25% cholesterol, and membrane proteins are increasingly resolved to contain conserved binding sites for cholesterol, CHS and other derivatives ^25– 29^. The natural 20–25% cholesterol habitat proved possible to be re-established by using DDM/CHS that forms wide bicelle-like micelles around membrane proteins, and greatly enhances GPCR activity and stability from just DDM ^25, 30^. Although open to improvement, DDM/CHS emerges as a viable method to unify solution-phase means—crystallography, electron microscopy, structural proteomics and nuclear magnetic resonance (NMR)—to spearhead charting the solution-phase structures of GPCRs and complexes. Such actionable atomic clarity is in urgent need and provides the pivotal foundation to further understand interactions with molecules, such as certain lipids. Although CHAPSO/DMPC bicelle has produced membrane protein crystals and NMR results ^31, 32^, the 2010 protocol is cautious and chooses not to present proteins in CHAPSO/DMPC for multiple reasons, as specified below and in Supplementary File 1, and increasing evidence since 2010 supports these cautions. NMR appears to favor zwitterionic CHAPSO/DMPC for technical convenience ^33– 35^, but mass spectrometry-based structural proteomics approaches are free from such technical constraints.

**Figure 2.**
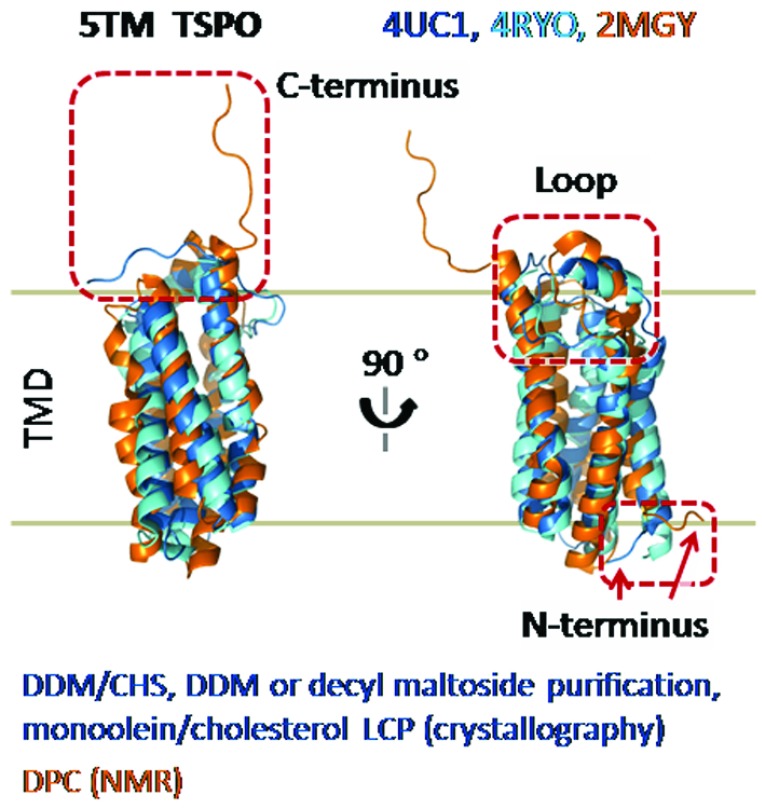
The 100% quaternary ammonium head groups of CHAPSO/DMPC call for cautions: structure alignment shows 100% DPC imposed variations to 5TM TSPO conformation. Blue or cyan, structures from two independent crystallizations in monoolein/cholesterol LCP (4UC1 or 4RYO); orange, structure from DPC-micelle NMR (2MGY). Distortions in all three domains of 5TM TSPO were seen in DPC-produced NMR structure (2MGY), contrasting the well-aligned independently acquired crystal structures from LCP (4UC1 and 4RYO) or DDM micelle and EM structure (not shown) ^62, 63^. TSPO structures were directly aligned in PyMOL.

The chemical structures of lipids matter. Indeed CHAPSO/DMPC presents a lipid-rich environment, but by no means resembles human GPCRs’ native lipid bilayer habitats. In-depth consideration of lipids is essential ( Figure 2) and is detailed in Supplementary File 1 and briefly summarized here. First, as a tool to present human GPCRs and complexes in near-native states (Step I, Figure 1), bilayer reconstitution is not restrained to 14:0/14:0 DMPC. CHAPSO/DMPC differs vastly from GPCR native lipid bilayer habitats in chemistry, and shall not dictate the choice of lipids to recreate bilayers. Neither does CHAPSO/DMPC bring much technical advantage to Step I for HDX-proteomics and most other solution-phase biophysical methods. To the contrary, the broad adaptability of downstream proteomics readout allows Step I to maximally prioritize protein structures, such as using various micelle, bicelle, bilayer, pellet, nanodisc or cell organelles ( Figure 1). Second, as a tool to solubilize GPCR for HDX-grade digestion and peptide-centric label analysis (Step II, Figure 1), the compatibility of high-dose CHAPSO/DMPC with large-scale direct HPLC MS and MS/MS runs appears controversial. By contrast, DDM alone with optional low TCEP proves effective and well suited for RP-HPLC MS instruments when applied rationally (further cross-discipline pitfalls discussed below), thus HDX-grade GPCR digestion (Step II) is no longer limited by solubilization. As a structural approach, HDX-proteomics choice of experimental conditions should let structure lead (Step I) and digestion follow (Step II). Touting CHAPSO/DMPC specifically for the HDX-proteomics approach—by arguing CHAPSO/DMPC is a better solubilization method than DDM for GPCR digestion based on questionable practices, yet minimizing structural considerations on proteins—is misleading.

Therefore, lipid choice in bilayer reconstitution is unrestricted to just CHAPSO/DMPC, NMR-favored zwitterionic head groups, or 14:0/14:0 chains, but should and could prioritize protein conformation, activity and stability. Indeed proteins may differ, and extensive method optimization is necessary. Membrane proteins’ responses to bilayer environment can be highly dynamic, diverse and sensitive; thus, multifaceted structure-activity measurements are essential to data interpretation. Recent rigorous bilayer reconstitutions for activity measurement typically examined various phospholipid head groups, chain lengths and cholesterol additive ^36– 38^, and increasingly chose POPS ^38^, POPE ^27^, POPE/POPG ^39– 41^, POPC/POPE/POPG ^27^ or DOPE/POPC/POPS ^40^ mixtures, with 16:0/18:1 or 18:1/18:1 fatty acid chains ^37, 40^, rather than 100% 14:0/14:0 DMPC.

## 4. Optimization of pepsin column reaction is a key for coverage

In the chosen digestion buffer, HDX proteolysis is completed within seconds of column residence time: the highly reactive pepsin column is obviously the most sensitive component of the platform to affect coverage and reproducibility. Pepsin column length, diameter, manufacturing of beads and column, temperature and flow rate may all change digestion products’ peptide length and reproducibility. Particularly, the pepsin-beads coupling reaction conditions affect pepsin surface density, activity and extent of autolysis—thus the effective enzyme surface concentration of final columns—and may vary between operators and manufacturers.

Because the 2010 HDX method is a completely automated protocol that integrates all experimental conditions, such as HDX incubation time, pepsin column flow rate, HPLC gradients and MS methods, manual operation only involves placing samples in designated sample trays, and selecting whether to use or not use the additional long-hour incubation module. However, the typical shelf life of each batch of pepsin beads and columns for peptide reproducibility is only about 10 months at 4°C. Therefore, rigorous practice means at least checking the optimal digestion flow rate and temperature based on the batch of beads and columns in use. These parameter updates are allowed in the 2010 protocol by simply typing the numbers, without changing the programming for sample handling and data acquisition.

Instead of under-digestion, low TM coverage is often caused by pepsin over-digestion, and may be rescued by optimizing pepsin column flow rate and temperature, and by applying longer HPLC for MS/MS ^2^. Alternatively, longer TM peptides may be generated by reversible partial deactivation of the pepsin column, and by blocking TM substrate access with bulkier, tighter or more facial amphiphiles and lipids. 

During initial digestion method development, the 30-min incubation with one column volume of pepsin bead slurry is commonly used to predict whether the pepsin column can reach digestion completion at seconds scale. But, the bead slurry format falls behind in peptide reproducibility, so all actual HDX data acquisitions in this protocol use pepsin-column digestions at precisely controlled flow rates.

Besides these four major points, this article further emphasizes systematic considerations of the subtle yet critical effects on membrane protein stability as follows.

## 5. Na ^+^ or K ^+^ matters

To the structure-function of membrane proteins, especially GPCRs, Na ^+ ^and K ^+^ are not always inter-exchangeable: thus the measurement itself shall not introduce Na/K interference. To proteomics, mixing Na ^+^ and K ^+^ may cause adduct ion formation of both Na ^+^ and K ^+ ^with peptides and other components, complicating peptide MS spectra. Upon 2009, development of the DLT GPCR HDX protocol started with asking whether to use Na ^+^ or K ^+^ buffers, and chose Na ^+^ for all buffers (protein dilution, H/D-incubation and quench/digestion buffers) for multiple reasons. First, Na ^+^ and K ^+^ may differ in structural and functional effects on TM proteins. Previous projects used K ^+^-based buffers to purify physiological-state high-activity C *c*O because both mitochondria and *Rs* bacteria have high internal K ^+^, whereas Na ^+^ was empirically screened as a tool to aid crystallogenesis ^29, 42– 44^. Na ^+^ and K ^+^ affect C *c*O Ca ^2+^ binding differently ^45, 46^, though the exact actions remain obscure. For GPCRs, unique among all common cations, Na ^+^ was long-observed to act as a fast allosteric mediator itself and control agonist/antagonist-distinct GPCR activities, and the structural bases started to be resolved by crystallography ^26, 36, 47– 56^. Physiological levels of Na ^+^ (and Li ^+^ to less extent) favored opiate receptor binding with antagonists against agonists ^48– 50^; thus the predominant use of NaCl buffers in GPCR purification may partly account for the larger difficulty to obtain stable GPCR-agonist complex upfront, than GPCR-antagonist complex. Consistently, crystallography revealed that Na ^+^ also modulates the ion flux activity of TM G protein-gated K ^+^ channel (GIRK2) by binding at specific sites in its intracellular domain immediate to the TM domain interface ^57, 58^. Consistently using NaH _2_PO _4_ rather than KH _2_PO _4_ for the digestion buffer avoided fast side effects that may occur even within the short pre-digestion time window.

Second, avoiding Na ^+^/K ^+^ mixing also prevented forming both Na ^+^ and K ^+^-adduct ions, such ions may exponentially complicate MS spectra and increase the risk of interfering with useful peptide peaks for both MS/MS isolation and HDX MS. The recent open data search method revealed extensive formation of peptide-Na ^+^ adduct ions ^59^. Third, continuing with Na ^+ ^buffers provides a common ground for structure-function and dynamic-static structure correlations, as most other function and structure characterizations of GPCRs were performed in Na ^+^ buffers. Lastly, abrupt changes of chemicals may destabilize membrane proteins; thus the method design sought to achieve effects with minimal disturbance of upstream conditions. Likewise, when membrane protein solution uses KCl ^60^, the H/D labeling and digestion buffers should ideally switch to K ^+^ versions accordingly.

## 6. 0.1% + 0 does not equal 0.05% + 0.05%: the buffering effect on membrane protein stability

Abrupt changes in buffer concentration, particularly dilution with zero (or sub-cmc)-detergent or zero-electrolyte solutions, tend to immediately impact membrane proteins and cause destabilization and aggregation. To provide such buffering protection, ^~^3× cmc DDM was included in quench solution and proved to increase digestion coverage better than zero-detergent quench ^2^. Similarly, destabilization and aggregation were seen in soluble proteins, such as human peptidyl arginine deiminase, upon zero-electrolyte dilution (unusually high occurrence of bimodal peptide HDX isotope envelopes despite pre-HDX removal of visible aggregates), and were solved also by buffering (unpublished results by Xi Zhang and Patrick R. Griffin). Often GPCR-CHAPSO/DMPC bicelles were prepared by adding CHAPSO/DMPC to, not replacing, the original DDM protein solution; thus the GPCR-CHAPS/DMPC bicelle sample contained double doses of micelle/bicelle, and may present an unequal ground for shielding/buffering effects. High occurrence of bimodal peptide HDX profile could also be artifacts from non-optimized HPLC or MS settings ^61^.

## 7. Full automation facilitates both structural and analytical rigor of membrane protein HDX

The DLT digestion method enabled membrane proteins to be analyzed on a fully automated HDX platform that orchestrates continuous sample handling and analysis. Rather than a dispensable convenience, the DLT-enabled automated protocol presents special advantages to maximize the structural/analytical rigor and sensitivity for membrane proteins. First, it eliminated detrimental post-labeling freeze-thaws. Post-H/D-labeling freeze-thaws of H/D-bearing membrane proteins or peptides may not only distort their H/D-label profiles, but also destabilize/aggregate membrane proteins, which worsens digestion peptide reproducibility and structural coverage. Second, it precisely controlled the time and temperature during and after GPCR H/D labeling and digestion, presenting a robust level ground that is vital for large-scale sensitive rigorous comparison to precisely locate stimuli-caused conformation changes. Membrane proteins’ non-TM domains are often highly sensitive to ligand and protein interactions, their amide HDX dynamics can vary on a split-second scale, though HDX data recording often starts with seconds. Third, its random acquisition order and insertion of one or more blank buffer runs between every two protein samples minimized carryover, facilitating analytical rigor. Indeed the technical error bars of %D from quadruplicates were tiny throughout the GPCR HDX examination, peptides of the ^~^89% HDX coverage were well reproduced proving analytical robustness and presented structural validity ( 2 and unpublished study by Xi Zhang and Patrick R. Griffin, *et al.*). Such rigor risks being compromised when operators have to frequently see and manually freeze, thaw and transfer protein and peptide samples. This fully automated enclosed membrane protein protocol also offers facile rigor to measure the effects of various light and electromagnetic stimuli. The longer-hour HDX incubation could be useful to directly profile the membrane protein complex stability.

Previous reviewer(s) since Nov 2015 repeatedly claimed the necessity of including high-pressure digestion and ion-mobility peptide separation for membrane protein HDX, and insisted that GPCR solubilization in HDX is not solved and that automation belongs to the future. However, most GPCR samples that entered the HDX pipeline have indeed been solubilized, many already studied by crystallography, which requires not only GPCR solubilization but also mono-dispersion. Automation was decided to be critical for the membrane protein HDX rigor and was included in the GPCR HDX protocol since its invention in late 2009; thus automation has been a reality since then. This protocol solved the high-coverage GPCR HDX challenge without needing high pressure for digestion or ion mobility MS for peptide separation. These perspectives shall help operators to achieve high-quality applications of this protocol.

## Conclusions

These perspectives, from the original method development, clarify critical technical nuances and logical thinking for the GPCR bottom-up HDX proteomics approach. The DDM-low-TCEP method resolved the technical barrier of HDX-grade GPCR digestion, and showed 7TM GPCR structures can be robustly approachable with bottom-up HDX proteomics; thus GPCR HDX is no longer hampered by solubilization during the digestion step. For effective application, it helps to view the GPCR HDX experiments as two modules that allow different flexibility in choosing detergent tools and HPLC MS settings ( Figure 1). Systematically considering membrane protein conformation and stability throughout the pipeline is vital, because Na ^+^/K ^+^ mixing, zero-detergent shock, freeze-thaws and imprecise sample handling may all affect the structural and/or analytical rigor of GPCR HDX results. The 2010 89% HDX coverage was obtained with both structural and analytical rigor. HDX proteomics is a structural technology, its choice of experimental conditions should—and now could—let structure lead and digestion follow, not vice versa.

## Supplementary material

Supplementary Figure 1: Contrast of hGPCR β _2_AR coverage using 2010 DLT versus urea digestion method, showing that appropriate application of DDM method did not cause solubilization problem.

.

Supplementary Figure 2: Contrast of 285 kDa hGABA _A_R coverage using 2015 FDD DDM-based digestion method versus common brutal force, further confirming that DDM is effective in protein solubilization during digestion.

.

Supplementary File 1: CHAPSO/DMPC bicelle versus DDM/CHS bicelle-like micelle is not unique to HDX approach; lipid chemical structures matter.

## References

[1] Sweedler JV: Striving for Reproducible Science. *Anal Chem.* 2015;87(23):11603–11604. 10.1021/acs.analchem.5b04300 26574918

[2] Zhang X Chien EY Chalmers MJ: Dynamics of the beta2-adrenergic G-protein coupled receptor revealed by hydrogen-deuterium exchange. *Anal Chem.* 2010;82(3):1100–1108. 10.1021/ac902484p 20058880 PMC2829980

[3] Duc NM Du Y Thorsen TS: Effective application of bicelles for conformational analysis of G protein-coupled receptors by hydrogen/deuterium exchange mass spectrometry. *J Am Soc Mass Spectrom.* 2015;26(5):808–817. 10.1007/s13361-015-1083-4 25740347 PMC4727453

[4] Chung KY Rasmussen SG Liu T: Conformational changes in the G protein Gs induced by the β _2_ adrenergic receptor. *Nature.* 2011;477(7366):611–615. 10.1038/nature10488 21956331 PMC3448949

[5] Shukla AK Westfield GH Xiao K: Visualization of arrestin recruitment by a G-protein-coupled receptor. *Nature.* 2014;512(7513):218–222. 10.1038/nature13430 25043026 PMC4134437

[6] Li S Lee SY Chung KY: Conformational analysis of g protein-coupled receptor signaling by hydrogen/deuterium exchange mass spectrometry. *Methods Enzymol.* 2015;557:261–278. 10.1016/bs.mie.2014.12.004 25950969

[7] Xiao K Chung J Wall A: The power of mass spectrometry in structural characterization of GPCR signaling. *J Recept Signal Transduct Res.* 2015;35(3):213–219. 10.3109/10799893.2015.1072979 26459735

[8] Konermann L Pan J Liu YH: Hydrogen exchange mass spectrometry for studying protein structure and dynamics. *Chem Soc Rev.* 2011;40(3):1224–1234. 10.1039/c0cs00113a 21173980

[9] Forest E Rey M: Hydrogen exchange mass spectrometry of proteins. Fundamentals, methods and applications. Weis, D. D. (Ed.); John Wiley & Sons, Ltd UK.2016;279–294. 10.1002/9781118703748.ch16

[10] Dawaliby R Trubbia C Delporte C: Allosteric regulation of G protein-coupled receptor activity by phospholipids. *Nat Chem Biol.* 2016;12(1):35–9. 10.1038/nchembio.1960 26571351 PMC4718399

[11] Savas JN Stein BD Wu CC: Mass spectrometry accelerates membrane protein analysis. *Trends Biochem Sci.* 2011;36(7):388–396. 10.1016/j.tibs.2011.04.005 21616670 PMC3222592

[12] Whitelegge JP: Integral membrane proteins and bilayer proteomics. *Anal Chem.* 2013;85(5):2558–2568. 10.1021/ac303064a 23301778 PMC3664232

[13] Pirrone GF Iacob RE Engen JR: Applications of hydrogen/deuterium exchange MS from 2012 to 2014. *Anal Chem.* 2015;87(1):99–118. 10.1021/ac5040242 25398026 PMC4287169

[14] Zhang X: Less is More: Membrane Protein Digestion Beyond Urea-Trypsin Solution for Next-level Proteomics. *Mol Cell Proteomics.* 2015;14(9):2441–2453. 10.1074/mcp.R114.042572 26081834 PMC4563727

[15] Hamuro Y Coales SJ Molnar KS: Specificity of immobilized porcine pepsin in H/D exchange compatible conditions. *Rapid Commun Mass Spectrom.* 2008;22(7):1041–1046. 10.1002/rcm.3467 18327892

[16] Wang Y Kumar N Solt LA: Modulation of retinoic acid receptor-related orphan receptor alpha and gamma activity by 7-oxygenated sterol ligands. *J Biol Chem.* 2010;285(7):5013–5025. 10.1074/jbc.M109.080614 19965867 PMC2836105

[17] Venable JD Dong MQ Wohlschlegel J: Automated approach for quantitative analysis of complex peptide mixtures from tandem mass spectra. *Nat Methods.* 2004;1(1):39–45. 10.1038/nmeth705 15782151

[18] Gillet LC Navarro P Tate S: Targeted data extraction of the MS/MS spectra generated by data-independent acquisition: a new concept for consistent and accurate proteome analysis. *Mol Cell Proteomics.* 2012;11(6):O111.016717. 10.1074/mcp.O111.016717 22261725 PMC3433915

[19] Black WA Stocks BB Mellors JS: Utilizing Microchip Capillary Electrophoresis Electrospray Ionization for Hydrogen Exchange Mass Spectrometry. *Anal Chem.* 2015;87(12):6280–6287. 10.1021/acs.analchem.5b01179 25992468 PMC4470751

[20] Doerr A: DIA mass spectrometry. *Nat Meth.* 2015;12:35 10.1038/nmeth.3234

[21] Rey M Forest E Pelosi L: Exploring the conformational dynamics of the bovine ADP/ATP carrier in mitochondria. *Biochemistry.* 2012;51(48):9727–9735. 10.1021/bi300759x 23136955

[22] Hebling CM Morgan CR Stafford DW: Conformational analysis of membrane proteins in phospholipid bilayer nanodiscs by hydrogen exchange mass spectrometry. *Anal Chem.* 2010;82(13):5415–5419. 10.1021/ac100962c 20518534 PMC2895417

[23] Kulak NA Pichler G Paron I: Minimal, encapsulated proteomic-sample processing applied to copy-number estimation in eukaryotic cells. *Nat Methods.* 2014;11(3):319–324. 10.1038/nmeth.2834 24487582

[24] Garavito RM Ferguson-Miller S: Detergents as tools in membrane biochemistry. *J Biol Chem.* 2001;276(35):32403–32406. 10.1074/jbc.R100031200 11432878

[25] Cherezov V Rosenbaum DM Hanson MA: High-resolution crystal structure of an engineered human beta2-adrenergic G protein-coupled receptor. *Science.* 2007;318(5854):1258–1265. 10.1126/science.1150577 17962520 PMC2583103

[26] Liu W Chun E Thompson AA: Structural basis for allosteric regulation of GPCRs by sodium ions. *Science.* 2012;337(6091):232–236. 10.1126/science.1219218 22798613 PMC3399762

[27] Penmatsa A Wang KH Gouaux E: X-ray structure of dopamine transporter elucidates antidepressant mechanism. *Nature.* 2013;503(7474):85–90. 10.1038/nature12533 24037379 PMC3904663

[28] Wang KH Penmatsa A Gouaux E: Neurotransmitter and psychostimulant recognition by the dopamine transporter. *Nature.* 2015;521(7552):322–327. 10.1038/nature14431 25970245 PMC4469479

[29] Qin L Hiser C Mulichak A: Identification of conserved lipid/detergent-binding sites in a high-resolution structure of the membrane protein cytochrome c oxidase. *Proc Natl Acad Sci U S A.* 2006;103(44):16117–16122. 10.1073/pnas.0606149103 17050688 PMC1616942

[30] Thompson AA Liu JJ Chun E: GPCR stabilization using the bicelle-like architecture of mixed sterol-detergent micelles. *Methods.* 2011;55(4):310–317. 10.1016/j.ymeth.2011.10.011 22041719 PMC3264755

[31] Rasmussen SG Choi HJ Rosenbaum DM: Crystal structure of the human beta2 adrenergic G-protein-coupled receptor. *Nature.* 2007;450(7168):383–387. 10.1038/nature06325 17952055

[32] Ujwal R Bowie JU: Crystallizing membrane proteins using lipidic bicelles. *Methods.* 2011;55(4):337–341. 10.1016/j.ymeth.2011.09.020 21982781 PMC3264687

[33] Stroud RM: New tools in membrane protein determination. *F1000 Biol Rep.* 2011;3:8. 10.3410/B3-8 21655333 PMC3100781

[34] Durr UH Gildenberg M Ramamoorthy A: The magic of bicelles lights up membrane protein structure. *Chem Rev.* 2012;112(11):6054–6074. 10.1021/cr300061w 22920148 PMC3497859

[35] Durr UH Soong R Ramamoorthy A: When detergent meets bilayer: birth and coming of age of lipid bicelles. *Prog Nucl Magn Reson Spectrosc.* 2013;69:1–22. 10.1016/j.pnmrs.2013.01.001 23465641 PMC3741677

[36] Alexandrov AI Mileni M Chien EY: Microscale fluorescent thermal stability assay for membrane proteins. *Structure.* 2008;16(3):351–359. 10.1016/j.str.2008.02.004 18334210

[37] Hattori M Hibbs RE Gouaux E: A fluorescence-detection size-exclusion chromatography-based thermostability assay for membrane protein precrystallization screening. *Structure.* 2012;20(8):1293–1299. 10.1016/j.str.2012.06.009 22884106 PMC3441139

[38] Althoff T Hibbs RE Banerjee S: X-ray structures of GluCl in *apo* states reveal a gating mechanism of Cys-loop receptors. *Nature.* 2014;512(7514):333–337. 10.1038/nature13669 25143115 PMC4255919

[39] Brohawn SG del Mármol J MacKinnon R: Crystal structure of the human K2P TRAAK, a lipid- and mechano-sensitive K ^+^ ion channel. *Science.* 2012;335(6067):436–441. 10.1126/science.1213808 22282805 PMC3329120

[40] Wang W Whorton MR MacKinnon R: Quantitative analysis of mammalian GIRK2 channel regulation by G proteins, the signaling lipid PIP _2_ and Na ^+^ in a reconstituted system. *eLife.* 2014;3:e03671. 10.7554/eLife.03671 25049222 PMC4135351

[41] Hite RK Yuan P Li Z: Cryo-electron microscopy structure of the Slo2.2 Na ^+^-activated K ^+^ channel. *Nature.* 2015;527(7577):198–203. 10.1038/nature14958 26436452 PMC4886347

[42] Zhang XThesis (Ph D): Investigating the functional roles of lipids in membrane protein cytochrome c oxidase from Rhodobacter sphaeroides using mass spectrometry and lipid profile modification, Michigan State University, Chemistry and Biochemistry & Molecular Biology.2009.

[43] Zhang X Hiser C Tamot B: Combined genetic and metabolic manipulation of lipids in *Rhodobacter sphaeroides* reveals non-phospholipid substitutions in fully active cytochrome *c* oxidase. *Biochemistry.* 2011;50(19):3891–3902. 10.1021/bi1017039 21476580 PMC3097905

[44] Zhang X Tamot B Hiser C: Cardiolipin deficiency in *Rhodobacter sphaeroides* alters the lipid profile of membranes and of crystallized cytochrome oxidase, but structure and function are maintained. *Biochemistry.* 2011;50(19):3879–3890. 10.1021/bi101702c 21476578 PMC3097902

[45] Lee A Kirichenko A Vygodina T: Ca ^2+^-binding site in *Rhodobacter sphaeroides* cytochrome *C* oxidase. *Biochemistry.* 2002;41(28):8886–8898. 10.1021/bi020183x 12102631

[46] Vygodina TV Kirichenko A Konstantinov AA: Cation binding site of cytochrome *c* oxidase: progress report. *Biochim Biophys Acta.* 2014;1837(7):1188–1195. 10.1016/j.bbabio.2014.02.025 24607866

[47] Bennett JP Jr Logan WJ Snyder SH: Amino acid neurotransmitter candidates: sodium-dependent high-affinity uptake by unique synaptosomal fractions. *Science.* 1972;178(4064):997–999. 10.1126/science.178.4064.997 4343557

[48] Pert CB Pasternak G Snyder SH: Opiate agonists and antagonists discriminated by receptor binding in brain. *Science.* 1973;182(4119):1359–1361. 10.1126/science.182.4119.1359 4128222

[49] Pert CB Snyder SH: Opiate Receptor Binding of Agonists and Antagonists Affected Differentially by Sodium. *Mol Pharmacol.* 1974;10:868–879. Reference Source

[50] Pasternak GW Snyder SH: Identification of novel high affinity opiate receptor binding in rat brain. *Nature.* 1975;253(5492):563–565. 10.1038/253563a0 1117990

[51] Kuhar MJ Pert CB Snyder SH: Regional distribution of opiate receptor binding in monkey and human brain. *Nature.* 1973;245(5426):447–450. 10.1038/245447a0 4127185

[52] Wilson HA Pasternak GW Snyder SH: Differentiation of opiate agonist and antagonist receptor binding by protein modifying reagents. *Nature.* 1975;253(5491):448–450. 10.1038/253448a0 46111

[53] Snyder SH Pasternak GW: Historical review: Opioid receptors. *Trends Pharmacol Sci.* 2003;24(4):198–205. 10.1016/S0165-6147(03)00066-X 12707007

[54] Horstman DA Brandon S Wilson AL: An aspartate conserved among G-protein receptors confers allosteric regulation of alpha 2-adrenergic receptors by sodium. *J Biol Chem.* 1990;265(35):21590–21595. 2174879

[55] Fenalti G Giguere PM Katritch V: Molecular control of δ-opioid receptor signalling. *Nature.* 2014;506(7487):191–196. 10.1038/nature12944 24413399 PMC3931418

[56] Katritch V Fenalti G Abola EE: Allosteric sodium in class A GPCR signaling. *Trends Biochem Sci.* 2014;39(5):233–244. 10.1016/j.tibs.2014.03.002 24767681 PMC4106411

[57] Whorton MR MacKinnon R: Crystal structure of the mammalian GIRK2 K ^+^ channel and gating regulation by G proteins, PIP _2_, and sodium. *Cell.* 2011;147(1):199–208. 10.1016/j.cell.2011.07.046 21962516 PMC3243363

[58] Whorton MR MacKinnon R: X-ray structure of the mammalian GIRK2-βγ G-protein complex. *Nature.* 2013;498(7453):190–197. 10.1038/nature12241 23739333 PMC4654628

[59] Chick JM Kolippakkam D Nusinow DP: A mass-tolerant database search identifies a large proportion of unassigned spectra in shotgun proteomics as modified peptides. *Nat Biotechnol.* 2015;33(7):743–749. 10.1038/nbt.3267 26076430 PMC4515955

[60] O'Connor C White KL Doncescu N: NMR structure and dynamics of the agonist dynorphin peptide bound to the human kappa opioid receptor. *Proc Natl Acad Sci U S A.* 2015;112(38):11852–11857. 10.1073/pnas.1510117112 26372966 PMC4586840

[61] Guttman M Wales TE Whittington D: Tuning a High Transmission Ion Guide to Prevent Gas-Phase Proton Exchange During H/D Exchange MS Analysis. *J Am Soc Mass Spectrom.* 2016;27(4):662–668. 10.1007/s13361-015-1330-8 26810432 PMC4829384

[62] Li F Liu J Zheng Y Garavito RM: Protein structure. Crystal structures of translocator protein (TSPO) and mutant mimic of a human polymorphism. *Science.* 2015;347(6221):555–558. 10.1126/science.1260590 25635101 PMC5125025

[63] Guo Y Kalathur RC Liu Q: Protein structure. Structure and activity of tryptophan-rich TSPO proteins. *Science.* 2015;347(6221):551–555. 10.1126/science.aaa1534 25635100 PMC4341906

