# Supplementary material for: Seven perspectives on GPCR H/D-exchange proteomics methods
Source: F1000Res. 2017 Jan 30;6:89. [Version 1] doi: 10.12688/f1000research.10667.1 (PMC5428523; doi:10.12688/f1000research.10667.1)
Supplement: Supplementary file 1 [file f1000research-6-11496-s0000.tgz › 0c3bab9d-a332-4b45-94d3-eaf3ddea60ca.pdf]

A. Previous urea-pepsin column

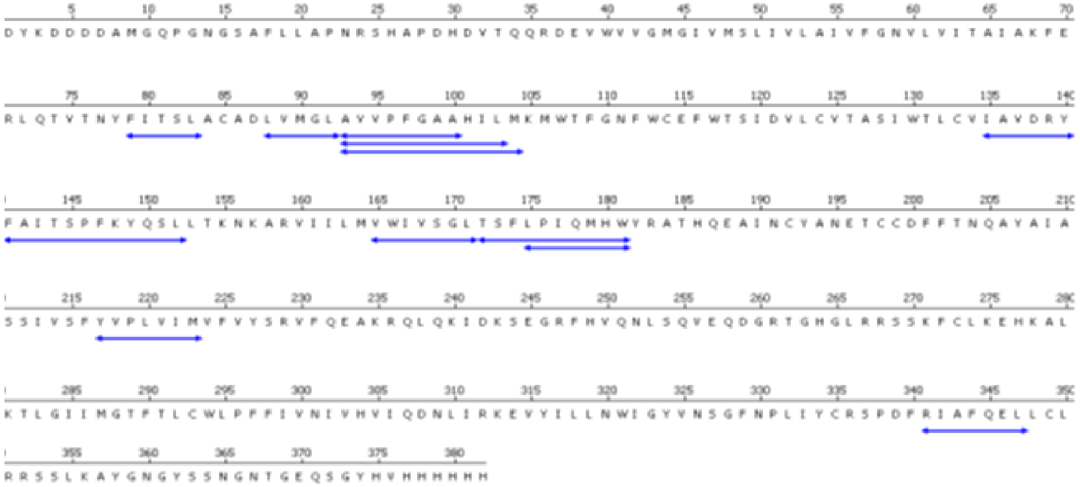

B. 2010 DDM-low-TCEP (DLT) pepsin-column (~90% MS/MS)

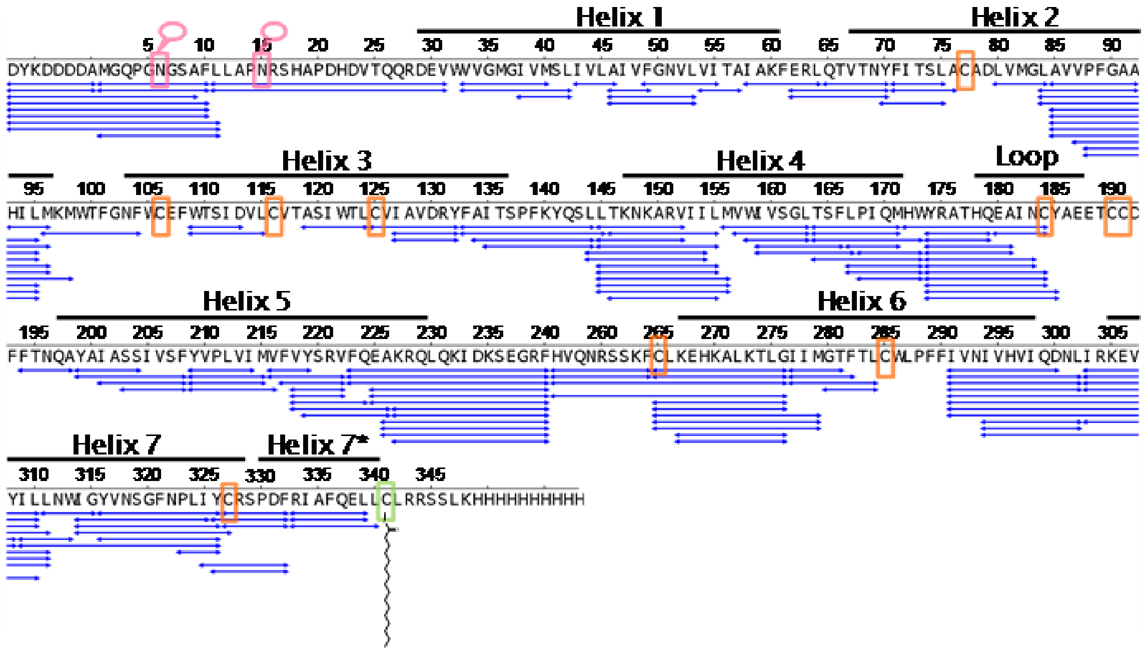

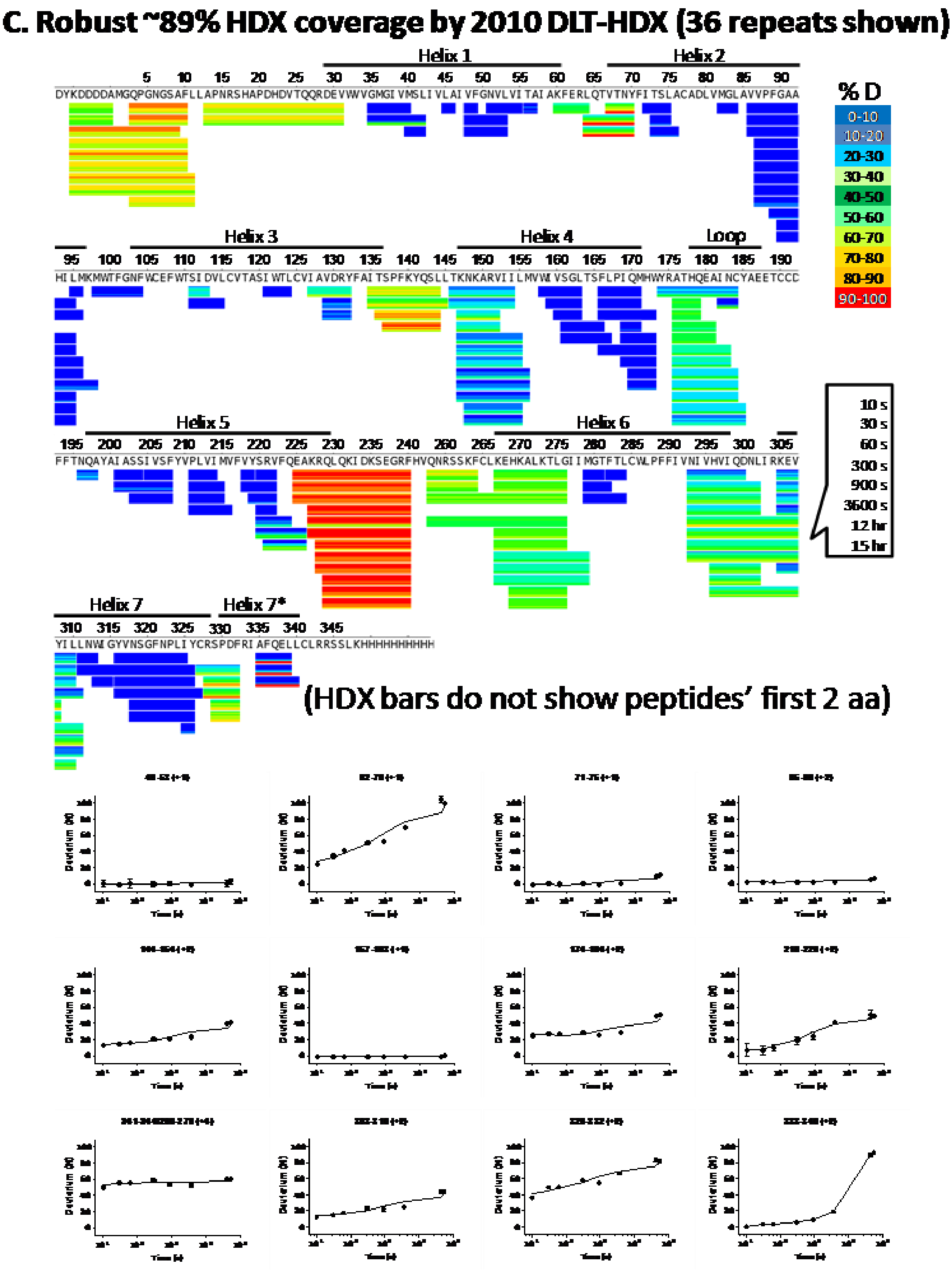

Supplementary Figure 1: Contrast of hGPCR β$_2$AR coverage using 2010 DLT versus urea digestion method, showing that appropriate application of DDM method did not cause solubilization problem. (**A**) Previous low coverage using urea-pepsin column digestions. Few peptides could be reproduced. Previous efforts also tried with failure: urea-trypsin solution -

/+ pre-digestion PNGase F solution (24% coverage, 8 peptides; 20%, 7 peptides), combined soluble and immobilized pepsin, combined pepsin and fungal XIII, combined pepsin and fungal XVIII, MeOH mobile phase, and isopropanol mobile phase (target sequence search); noticed many non-GPCR-related proteins identified by trypsin (human proteome search). (**B**) 2010 DLT ~90% MS/MS coverage and (**C**) reproducible ~89% coverage for HDX. Each HDX peptide was reproduced by hundreds of independent digestions. Part of this figure was originally published in *Anal Chem* [43]. Adapted with permission from Xi Zhang *et al*, *Anal Chem* 2010 82 1100-1108, © 2010 American Chemical Society.
