# Supplementary material for: Seven perspectives on GPCR H/D-exchange proteomics methods
Source: F1000Res. 2017 Jan 30;6:89. [Version 1] doi: 10.12688/f1000research.10667.1 (PMC5428523; doi:10.12688/f1000research.10667.1)
Supplement: Supplementary file 2 [file f1000research-6-11496-s0001.tgz › 7dc0c4df-aeec-425a-be81-c498b59fb6fe.pdf]

A. Typical proteomic sequencing of purified (a1)2(b3)3 $GABA_AR$

Results

1. Identified a few peptides for chicken and human b1 subunits, which were not expressed in the human a1b3 $GABA_AR$ sample.
2. Manually arbitrarily increased peptide coverage for human b3 subunit.
3. Coverage after manual adjustments:
   - bovine GBRA1: 13 peptides, 28.3% coverage, FLAG tag not covered.
   - human GBRB3: 7 peptides, 15.8%.
   - human 5HT3R: 2 peptides.
   - bovine ATPA1: 2 peptides.

Conclusions

Recommended to use new anti-FLAG resin for important preparations.
(Based on unpublished data provided by Dr. Keith W. Miller lab.)

B. 2015 FDD: <0.04 cell plates, <10 s digestion, minutes total preparation time for injection-ready

Up to 99% coverage, with PTM % occupancy

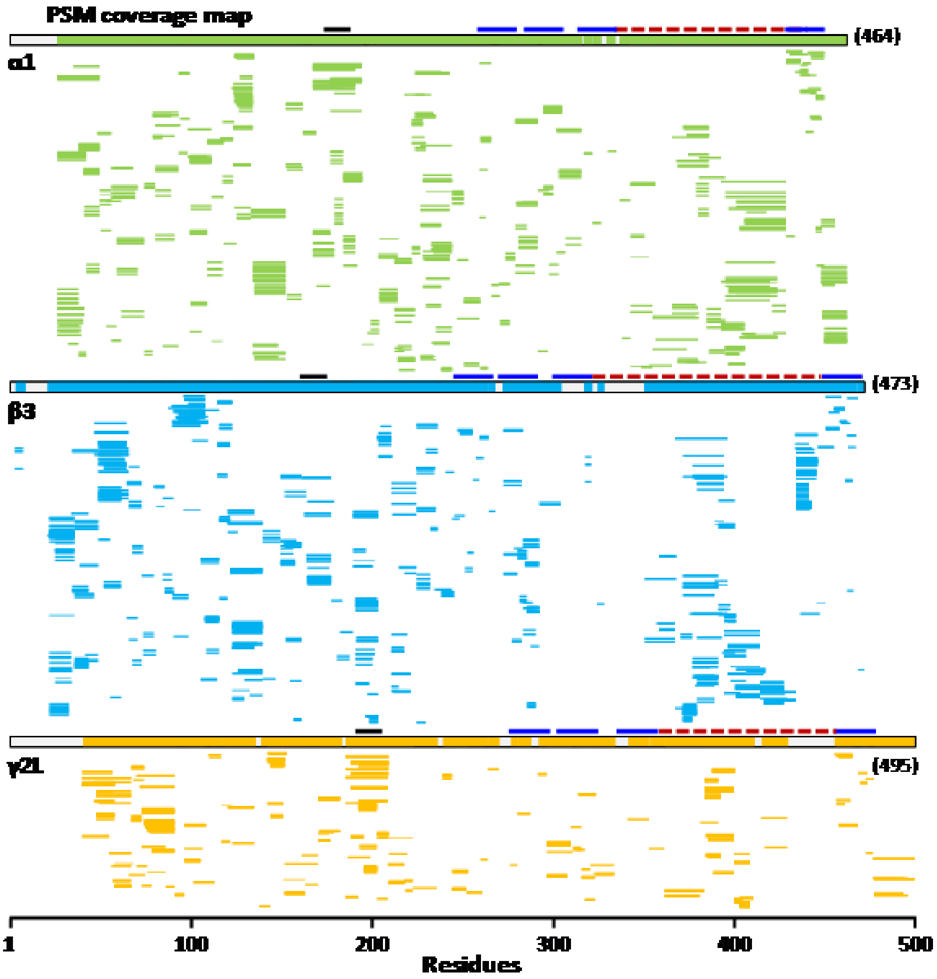

Supplementary Figure 2: Contrast of 285 kDa hGABA$_A$R coverage using 2015 FDD DDM-based digestion method versus common brutal force, further confirming that DDM is effective in protein solubilization during digestion. (**A**) Representative former proteomic sequencing results of purified human hGABA$_A$R using prevalent digestion method, after trying for years and consuming many batches of 60-plate purified proteins (small target sequence search and manually adjusted). Data provided by Dr. Keith W. Miller lab (Massachusetts General Hospital and Harvard Medical School). (**B**) 2015 FDD up to 99% coverage with PTM % occupancy, using <0.04 plate of cells, seconds-minutes, no brutal force (showing Fig. 4A of Ref [44] MCP 2015, adapted from Ref [45] MCP 2016). Part of this figure was originally published in *Mol Cell Proteomics*. Xi Zhang, *Mol Cell Proteomics* 2015 14 2441-2453, and Xi Zhang, *Mol Cell Proteomics* 2016 Apr12 Epub DOI M114.047514, © the American Society for Biochemistry and Molecular Biology (with permission).
