# Supplementary material for: Seven perspectives on GPCR H/D-exchange proteomics methods
Source: F1000Res. 2017 Jan 30;6:89. [Version 1] doi: 10.12688/f1000research.10667.1 (PMC5428523; doi:10.12688/f1000research.10667.1)
Supplement: Supplementary file 3 [file f1000research-6-11496-s0002.tgz › 115b7092-5adf-4cb4-aa5f-535f9ef06724.docx]

***Seven perspectives on GPCR H/D-exchange proteomics methods*** (Xi Zhang, PhD)

**Supplementary File 1: CHAPSO/DMPC bicelle vs DDM/CHS bicelle-like micelle is not unique to HDX approach; lipid chemical structures matter.**

The chemical structures of lipids matter. Indeed CHAPSO/DMPC presents a lipid-rich environment, but by no means resembles human GPCRs’ native lipid bilayer habitats. First, although overall zwitterionic, CHAPSO/DMPC presents a 100% composition of strong fixed positive-charged quaternary ammonium in head groups from both detergent and lipid on the bilayer surfaces, and contradicts the balanced natural lipid compositions. Mammalian GPCR natural membrane lipid reservoir can contain phosphatidylcholine (PC), phosphatidylethanolamine (PE), phosphatidylglycerol (PG), phosphatidylserine (PS), phosphatidic acid (PA), phosphatidylinositol (PI), PI phosphate (PIPn), sphingomyelin, sulfo-phospholipids, plasmalogens and cholesterols, most of which carry mildly-charged amine, polar nonionic, or negatively-charged groups in the head group moiety. This charge group variation can translate into different interactions with cations (Na^+^, K^+^, *etc*), anions (Cl¯), ligands, protein residues and other lipids at the bilayer surfaces.

Besides a balanced bilayer lipid reservoir, increasing evidence demonstrates membrane proteins are complexes with lipids, the composition of their tightly-associated lipids reflects the membrane bilayer and may fluctuate with purification conditions, but selectively enriches certain types of lipids—often against PC. For example, purified and crystallized active *Rhodobacter sphaeroides (Rs)* cytochrome *c* oxidase (C*c*O) enriched or maintained cardiolipin, PG, PE and other *Rs*-unique sulfoquinovosyl-, ornithine- and glutamine-lipids, but decreased PC level [1-3]. Consistently, crystallography combined with mass spectrometry also resolved 13 co-crystallized endogenous lipids in bovine tissue C*c*O, only one was PC [4]. Several crystallography studies resolved bound PG, cardiolipin, cholesterol and CHS (cell-endogenous or from purification and crystallization media) in various TM proteins [4-12], though the resolution of crystallography is often insufficient to unambiguously distinguish between PC and PE head groups. Although protein-dependent, currently most established function-important lipids for TM proteins include PE [13-15], PG-cardiolipin [1-3], or cholesterols [5-8, 12], but rarely PC. TM proteins may even display no affinity to PC [16]. So far little evidence shows human GPCRs predominantly enrich for PC, let alone to 100%. If a protein’s native habitat does not prefer PC, then forcing a 100% PC-head-group-like environment as a substitute may call for concerns.

Meanwhile, artificial supply of defined phospholipids to isolated TM proteins often found that the added lipids act as allosteric regulators in various facets of protein functions, and differ vastly with proteins and lipid head groups given the same chain lengths. This emphasizes empirical screening when choosing lipids for bilayer reconstitution, rather than staying confined to CHAPSO/DMPC. Contrasting PIPn [9, 10, 17-23] and PA [24] that emerged as drastic switches via direct site-specific binding to TM proteins, such as ion channels and transporters, PS, PC, PE, PG and sphingomyelin, appeared more modulatory, but nonetheless different. For example, POPS stabilized GPCR bovine rhodopsin-G_t_ complex and activated G_t_ more strongly than POPC and POPE [25]; under the same 14:0/14:0 chains and CHAPSO system, bicelles that contain PS and PA stabilized rhodopsin-G_t_ complex stronger than just PC [26]. When incubated with DDM-purified pentameric ion channel *C. elegans* GluCl/Sf9, POPS was also stronger than POPC in potentiating prototypic agonist binding, competing with ivermectin ligand, binding and stabilizing protein complex in solution, but weaker than POPC in facilitating high-resolution crystallogenesis [22, 27]. Under regular reconstitution procedure GluCl resented POPC incorporation; imposing POPC for four weeks ahead of crystallogenesis led to several resolved POPC bound to subunit interfaces of pentameric GluCl (with PC head group wedging between TM helices), and they altered protein structure [27]. Experiments using *in vitro* reconstituted PC-based bilayer also found that POPC changed K^+^ channel ion flux function differently from the group of POPE, POPS, POPG and cardiolipin [24], and PC head group bound—via electrostatic interactions—to K^+^ channel residues located at the bilayer surfaces in both extracellular and intracellular domains [28]. Therefore, although naturally not preferred by TM proteins, artificially added DMPC may perturb protein structure; the artificial 100% fixed positive-charged quaternary ammonium surface of CHAPSO/DMPC may particularly affect the conformations of GPCR’s extra- and intra-cellular domains besides TM helices.

Second, DMPC lipid fatty acid chain dimyristoyl 14:0/14:0 is not representative of mammalian GPCRs’ natural lipid habitat, but forms a much thinner bilayer. Lipid bicelle thickness in reconstitution can alter TM protein conformation and function [29], and these effects are larger on purified proteins than when lipids are introduced early as detergent-lipid buffers during solubilization-purification steps. Generally for TM protein stabilization, 14:0/14:0 lipids are starkly less effective than 16:0/18:1 (PO, 1-palmitoyl-2-oleoyl) and 18:1/18:1 (DO, 1,2-dioleoyl) versions [22].

Third, CHAPS, chemically similar to CHAPSO, damages TM human gamma-aminobutyric acid type A receptor activity by several folds compared with natural lipid bilayer and with DDM/CHS [30], and destabilizes other TM proteins, such as eukaryotic membrane protein J from Sf9, compared with DDM [31].

Furthermore, using DDM [32] or decyl maltoside [11] purification and direct DDM micelle [32] or 18:1 monoolein/cholesterol lipidic cubic phase (LCP) [11, 32] crystallization, recent crystallography and electron microscopy (EM) structures of a 5TM translocator protein (TSPO) revealed that, the 2014 NMR’s dodecyl phosphatidylcholine (DPC) detergent micelle (100% fixed positive-charged head group, 12:0) [33] had distorted the protein conformation in several domains, and exposed charges on TM surface likely by disrupting H-bond network [11, 32] (**Figure 2**, in main text). Although membrane proteins each differ, 7TM human GPCRs are unlikely to completely escape such strong effects of 100% fixed positive-charged head groups throughout the bilayer, which can be empirically examined. Consistently, DDM/CHS purification coupled with monoolein/cholesterol LCP routinely generated crystals that resolved the GPCR β_2_AR construct’s near-full structure at high resolution (PDB 2RH1, 2.4 Å, 2007, T4L fusion), and, regardless of using various covalent fusion tools or non-covalent antibody stabilization, this method and its maltose-neopentyl glycol/CHS variation have robustly reproduced high-quality crystals for nearly all the other human GPCRs resolved to date [6, 34-38]. By contrast, despite extensive optimization and antibody stabilization, CHAPSO/DMPC bicelle remains unable to resolve the extracellular domain and the upper half of the TM domain (PDB 2R4R, 3.5/3.7 Å, 2007, Fab), leaving these domains unavailable for comparison to further refine the effects of CHAPSO/DMPC (**Figure S1**). Nonetheless this observation itself alarms that CHAPSO/DMPC bicelle might have limited capacity to stabilize the GPCR protein. For LeuT, a bacterial ortholog of TM neurotransmitter sodium symporter, the CHAPSO/DMPC-crystallized DDM-purified structures highly resembled the n-octyl-β-D-glucopyranoside-crystallized version, but differed slightly in the loops at the TM domain interface or bilayer surface [39]. Together, these results highlight that membrane proteins’ responses to bilayer environment can be highly dynamic, diverse and sensitive, thus multifaceted structure-activity measurements are essential to data interpretation.

Consistently, a study published after the November 2015 pre-submission inquiry of this manuscript reported the following: the human β_2_AR expressed and purified from Sf9 cells enriched cholesterol by 17.7 fold and PG by 4.4 fold (from membrane as a percentage of total lipids), contrasting only 2.2 fold for PC, 1.2 fold for PE, and 0.1, 0.3, 0.3 (depletion) for PS, PI, sphingomyelin respectively [40]. The extraction efficiency of tightly-bound endogenous lipids from detergent-purified proteins was undisclosed. β_2_AR also enriched 18:1/18:1 chains by 80 fold, 16:1/18:1 by 8.6 fold, and reported no 14:0/14:0. Its lipid modulation assay has excluded the 14:0/14:0 DMPC [40]. These results argue against using 100% DMPC and support adding CHS to prepare native protein buffer.

Recent rigorous bilayer reconstitutions for activity measurement typically examined various phospholipid head groups, chain lengths and cholesterol additive [22, 27, 41], and increasingly chose POPS [27], POPE [12], POPE/POPG [17, 20, 42], POPC/POPE/POPG [12] or DOPE/POPC/POPS [20] mixtures, with 16:0/18:1 or 18:1/18:1 fatty acid chains [20, 22], rather than 100% 14:0/14:0 DMPC.

In conclusion, as a tool to present human GPCRs and complexes in near-native states (Step I, **Figure 1**, in main text), the lipid choice in bilayer reconstitution is not restrained to 14:0/14:0 DMPC, NMR-favored zwitterionic head groups, or 14:0/14:0 chains.

**Figure S1:** **CHAPSO/DMPC may raise structural concerns for human GPCR: carazolol-bound 7TM hGPCR β_2_AR structures resolved by crystallography from different media.** Blue (left), structure from DDM/CHS purification and monoolein/cholesterol lipidic cubic phase (LCP) crystallization (2RH1, 2.4 Å, β_2_AR with T4L fusion). Orange (right), structure from CHAPSO/DMPC-bicelle crystallization (2R4R, 3.5/3.7 Å, β_2_AR with ICL3-Fab5). ECD of 2R4R was unresolved, and thus unavailable for comparison. 2RH1 and 2R4R were aligned in PyMOL by selecting only those residues resolved in 2R4R.

**References**

[1] Zhang, X. Thesis (Ph D). Investigating the functional roles of lipids in membrane protein cytochrome c oxidase from Rhodobacter sphaeroides using mass spectrometry and lipid profile modification, Michigan State University, Chemistry and Biochemistry & Molecular Biology, 2009.

[2] Zhang, X., Tamot, B., Hiser, C., Reid, G. E.*, et al.*, Cardiolipin deficiency in Rhodobacter sphaeroides alters the lipid profile of membranes and of crystallized cytochrome oxidase, but structure and function are maintained. *Biochemistry* 2011, *50*, 3879-3890.

[3] Zhang, X., Hiser, C., Tamot, B., Benning, C.*, et al.*, Combined genetic and metabolic manipulation of lipids in Rhodobacter sphaeroides reveals non-phospholipid substitutions in fully active cytochrome c oxidase. *Biochemistry* 2011, *50*, 3891-3902.

[4] Shinzawa-Itoh, K., Aoyama, H., Muramoto, K., Terada, H.*, et al.*, Structures and physiological roles of 13 integral lipids of bovine heart cytochrome c oxidase. *EMBO J* 2007, *26*, 1713-1725.

[5] Cherezov, V., Rosenbaum, D. M., Hanson, M. A., Rasmussen, S. G.*, et al.*, High-resolution crystal structure of an engineered human beta2-adrenergic G protein-coupled receptor. *Science* 2007, *318*, 1258-1265.

[6] Liu, W., Chun, E., Thompson, A. A., Chubukov, P.*, et al.*, Structural basis for allosteric regulation of GPCRs by sodium ions. *Science* 2012, *337*, 232-236.

[7] Wang, K. H., Penmatsa, A., Gouaux, E., Neurotransmitter and psychostimulant recognition by the dopamine transporter. *Nature* 2015, *521*, 322-327.

[8] Qin, L., Hiser, C., Mulichak, A., Garavito, R. M., Ferguson-Miller, S. M., Identification of conserved lipid/detergent-binding sites in a high-resolution structure of the membrane protein cytochrome c oxidase. *Proc Natl Acad Sci U S A* 2006, *103*, 16117-16122.

[9] Whorton, M. R., MacKinnon, R., Crystal structure of the mammalian GIRK2 K+ channel and gating regulation by G proteins, PIP2, and sodium. *Cell* 2011, *147*, 199-208.

[10] Hansen, S. B., Tao, X., MacKinnon, R., Structural basis of PIP2 activation of the classical inward rectifier K+ channel Kir2.2. *Nature* 2011, *477*, 495-498.

[11] Li, F., Liu, J., Zheng, Y., Garavito, R. M., Ferguson-Miller, S. M., Protein structure. Crystal structures of translocator protein (TSPO) and mutant mimic of a human polymorphism. *Science* 2015, *347*, 555-558.

[12] Penmatsa, A., Wang, K. H., Gouaux, E., X-ray structure of dopamine transporter elucidates antidepressant mechanism. *Nature* 2013, *503*, 85-90.

[13] Wang, X., Bogdanov, M., Dowhan, W., Topology of polytopic membrane protein subdomains is dictated by membrane phospholipid composition. *EMBO J* 2002, *21*, 5673-5681.

[14] Bogdanov, M., Heacock, P. N., Dowhan, W., A polytopic membrane protein displays a reversible topology dependent on membrane lipid composition. *EMBO J* 2002, *21*, 2107-2116.

[15] Bogdanov, M., Dowhan, W., Phospholipid-assisted protein folding: phosphatidylethanolamine is required at a late step of the conformational maturation of the polytopic membrane protein lactose permease. *EMBO J* 1998, *17*, 5255-5264.

[16] Jansen, F., Kalbe, B., Scholz, P., Fraenzel, B.*, et al.*, Biochemical large-scale interaction analysis of murine olfactory receptors and associated signaling proteins with PDZ domains. *Mol Cell Proteomics* 2015, Epub May 15.

[17] Brohawn, S. G., del Marmol, J., MacKinnon, R., Crystal structure of the human K2P TRAAK, a lipid- and mechano-sensitive K+ ion channel. *Science* 2012, *335*, 436-441.

[18] Brohawn, S. G., Campbell, E. B., MacKinnon, R., Physical mechanism for gating and mechanosensitivity of the human TRAAK K+ channel. *Nature* 2014, *516*, 126-130.

[19] Brohawn, S. G., Su, Z., MacKinnon, R., Mechanosensitivity is mediated directly by the lipid membrane in TRAAK and TREK1 K+ channels. *Proc Natl Acad Sci U S A* 2014, *111*, 3614-3619.

[20] Wang, W., Whorton, M. R., MacKinnon, R., Quantitative analysis of mammalian GIRK2 channel regulation by G proteins, the signaling lipid PIP2 and Na+ in a reconstituted system. *Elife* 2014, *3*, e03671.

[21] Hansen, S. B., Lipid agonism: The PIP paradigm of ligand-gated ion channels. *Biochim Biophys Acta* 2015, *1851*, 620-628.

[22] Hattori, M., Hibbs, R. E., Gouaux, E., A fluorescence-detection size-exclusion chromatography-based thermostability assay for membrane protein precrystallization screening. *Structure* 2012, *20*, 1293-1299.

[23] Whorton, M. R., MacKinnon, R., X-ray structure of the mammalian GIRK2-betagamma G-protein complex. *Nature* 2013, *498*, 190-197.

[24] Hite, R. K., Butterwick, J. A., MacKinnon, R., Phosphatidic acid modulation of Kv channel voltage sensor function. *Elife* 2014, *3*, e04366.

[25] Jastrzebska, B., Goc, A., Golczak, M., Palczewski, K., Phospholipids are needed for the proper formation, stability, and function of the photoactivated rhodopsin-transducin complex. *Biochemistry* 2009, *48*, 5159-5170.

[26] Kaya, A. I., Thaker, T. M., Preininger, A. M., Iverson, T. M., Hamm, H. E., Coupling efficiency of rhodopsin and transducin in bicelles. *Biochemistry* 2011, *50*, 3193-3203.

[27] Althoff, T., Hibbs, R. E., Banerjee, S., Gouaux, E., X-ray structures of GluCl in apo states reveal a gating mechanism of Cys-loop receptors. *Nature* 2014, *512*, 333-337.

[28] Butterwick, J. A., MacKinnon, R., Solution structure and phospholipid interactions of the isolated voltage-sensor domain from KvAP. *J Mol Biol* 2010, *403*, 591-606.

[29] daCosta, C. J., Dey, L., Therien, J. P., Baenziger, J. E., A distinct mechanism for activating uncoupled nicotinic acetylcholine receptors. *Nat Chem Biol* 2013, *9*, 701-707.

[30] Zhang, X., Miller, K. W., Dodecyl maltopyranoside enabled purification of active human GABA type A receptors for deep and direct proteomic sequencing. *Mol Cell Proteomics* 2015, *14*, 724-738.

[31] Kawate, T., Gouaux, E., Fluorescence-detection size-exclusion chromatography for precrystallization screening of integral membrane proteins. *Structure* 2006, *14*, 673-681.

[32] Guo, Y., Kalathur, R. C., Liu, Q., Kloss, B.*, et al.*, Protein structure. Structure and activity of tryptophan-rich TSPO proteins. *Science* 2015, *347*, 551-555.

[33] Jaremko, L., Jaremko, M., Giller, K., Becker, S., Zweckstetter, M., Structure of the mitochondrial translocator protein in complex with a diagnostic ligand. *Science* 2014, *343*, 1363-1366.

[34] Chun, E., Thompson, A. A., Liu, W., Roth, C. B.*, et al.*, Fusion partner toolchest for the stabilization and crystallization of G protein-coupled receptors. *Structure* 2012, *20*, 967-976.

[35] Qin, L., Kufareva, I., Holden, L. G., Wang, C.*, et al.*, Structural biology. Crystal structure of the chemokine receptor CXCR4 in complex with a viral chemokine. *Science* 2015, *347*, 1117-1122.

[36] Fenalti, G., Giguere, P. M., Katritch, V., Huang, X. P.*, et al.*, Molecular control of delta-opioid receptor signalling. *Nature* 2014, *506*, 191-196.

[37] Fenalti, G., Zatsepin, N. A., Betti, C., Giguere, P.*, et al.*, Structural basis for bifunctional peptide recognition at human delta-opioid receptor. *Nat Struct Mol Biol* 2015, *22*, 265-268.

[38] Huang, W., Manglik, A., Venkatakrishnan, A. J., Laeremans, T.*, et al.*, Structural insights into micro-opioid receptor activation. *Nature* 2015, *524*, 315-321.

[39] Wang, H., Elferich, J., Gouaux, E., Structures of LeuT in bicelles define conformation and substrate binding in a membrane-like context. *Nat Struct Mol Biol* 2012, *19*, 212-219.

[40] Dawaliby, R., Trubbia, C., Delporte, C., Masureel, M.*, et al.*, Allosteric regulation of G protein-coupled receptor activity by phospholipids. *Nat Chem Biol* 2015, Epub Nov 16, DOI: 10.1038/nchembio.1960.

[41] Alexandrov, A. I., Mileni, M., Chien, E. Y., Hanson, M. A., Stevens, R. C., Microscale fluorescent thermal stability assay for membrane proteins. *Structure* 2008, *16*, 351-359.

[42] Hite, R. K., Yuan, P., Li, Z., Hsuing, Y.*, et al.*, Cryo-electron microscopy structure of the Slo2.2 Na-activated K channel. *Nature* 2015, *527*, 198-203.

[43] Zhang, X., Chien, E. Y., Chalmers, M. J., Pascal, B. D.*, et al.*, Dynamics of the beta2-adrenergic G-protein coupled receptor revealed by hydrogen-deuterium exchange. *Anal Chem* 2010, *82*, 1100-1108.

[44] Zhang, X., Less is more: Membrane protein digestion beyond urea-trypsin solution for next-level proteomics. *Mol Cell Proteomics* 2015, *14*, 2441-2453.

[45] Zhang, X., Instant integrated ultradeep quantitative-structural membrane proteomics discovered PTM signatures for human Cys-loop receptor subunit bias. *Mol Cell Proteomics* 2016, Epub Apr 12, DOI M114.047514.
