# Supplementary material for: Seven perspectives on GPCR H/D-exchange proteomics methods
Source: F1000Res. 2017 Jan 30;6:89. [Version 1] doi: 10.12688/f1000research.10667.1 (PMC5428523; doi:10.12688/f1000research.10667.1)
Supplement: Peer review history

# Peer review: Referee response for version 1

**DOI:** 10.5256/f1000research.11496.r22463

**Published:** 2017-05-08

**Authors:** Jun Qin, Chenxi Jia

**Affiliations:** 1 State Key Laboratory of Proteomics, Beijing Proteome Research Center (BPRC), Beijing Institute of Radiation Medicine, National Center for Protein Sciences (PHOENIX Center), Beijing, China; 2 State Key Laboratory of Proteomics, Beijing Proteomics Research Center, Beijing Institute of Radiation Medicine, National Center for Protein Sciences (PHOENIX Center), Beijing, China

This manuscript presented seven perspectives for investigation of GPCR structure using H/D-exchange with mass spectrometry. The manuscript was well-written and clarified many misconceptions in using H/D exchange in getting GPCR rough structure information. It is nice that the author put emphasis in the description of how to maintain the solution structure of GPCR with its relevant lipid environments during the H/D exchange reaction. It can be accepted after further addressing the following questions.

 1). HDX can be used to illustrate the effect of post-translational modifications ^1^ on protein dynamics, ligand binding, and substrate specificity. It is an important part of application of this intriguing technology and should be included in this manuscript.

 2). Crosslinking (XL) mass spectrometry of GPCR in its native state (with proper lipids and solubilizing detergents) may be a rival to HDX in the future. XL may be used with endogenous GPCRs. This should be discussed.

 3).The manuscript need to be further proof-read. There are many typos and grammar errors.

 One example:

 (Page 4, Line 25) “How the presentation methods” should be corrected as “how the present methods”.

We have read this submission. We believe that we have an appropriate level of expertise to confirm that it is of an acceptable scientific standard.

## References

[1] ZhouBZhangZY: Application of hydrogen/deuterium exchange mass spectrometry to study protein tyrosine phosphatase dynamics, ligand binding, and substrate specificity. *Methods*.2007;42(3) : 10.1016/j.ymeth.2007.02.020 227-33 10.1016/j.ymeth.2007.02.020 17532509PMC2001256

# Peer review: Referee response for version 1

**DOI:** 10.5256/f1000research.11496.r21854

**Published:** 2017-04-20

**Authors:** Dapeng Chen

**Affiliations:** 1 Department of Chemistry & Biochemistry, University of Maryland, College Park, MD, 20740, USA

The coupling of amide hydrogen/deuterium exchange (HDX) with mass spectrometry has been successfully used for the determination of protein dynamics. Recent studies ^1^^-^^2^ have shown that this method can reach high protein sequence coverage (>80%) in the study of GPCRs. However, frustrations exist, such as the confusion of the HPLC method and the inconsistency of the use of detergents. In the opinion article, Zhang discussed three critical technical aspects that should be carefully examined including 1) bottom-up MS analysis, 2) the use of detergents, and 3) the proteolysis strategy. Particularly, the author expanded the discussion on the problematic use of CHAPSO/DMPC for the membrane protein solution. The author claimed that this solution could form positively charged ions and interfere with MS analysis. In addition, the author raised their concerns of the presence of Na ^+^ and K ^+^ in the buffer, which could encourage the production of ion-added peptides and hinder MS data interpretation.

 It is an undeniable fact that the DIA MS/MS strategy has been highly successful in bottom-up proteomics analysis. However, the application of this method could be very challenging due to the limitation of the development of bioinformatics tools. Therefore, the author’s statement that DIA could improve MS/MS analysis independently is open to argument. Nevertheless, the combination of DDA and DIA is preferable, which would further improve sequence coverage in bottom-up proteomics analysis.

 Recently, there have been advancements in top-down proteomics analysis. GPCRs are low-mass proteins (~40 kDa) and it is feasible to obtain high resolution of MS/MS data when using the ETD fragmentation method. When coupled with bioinformatics tools, top-down proteomics analysis can be used to determine protein topology ^3^. The author may consider the potential use of top-down proteomics analysis on GPCRs.

I have read this submission. I believe that I have an appropriate level of expertise to confirm that it is of an acceptable scientific standard.

## References

[1] ZhangXChienEYChalmersMJPascalBDGatchalianJStevensRCGriffinPR: Dynamics of the beta2-adrenergic G-protein coupled receptor revealed by hydrogen-deuterium exchange. *Anal Chem*.2010;82(3) : 10.1021/ac902484p 1100-8 10.1021/ac902484p 20058880PMC2829980

[2] DucNMDuYThorsenTSLeeSYZhangCKatoHKobilkaBKChungKY: Effective application of bicelles for conformational analysis of G protein-coupled receptors by hydrogen/deuterium exchange mass spectrometry. *J Am Soc Mass Spectrom*.2015;26(5) : 10.1007/s13361-015-1083-4 808-17 10.1007/s13361-015-1083-4 25740347PMC4727453

[3] LeeAEGeis-AsteggianteLDixonEKKimYKashyapTRWangYFushmanDFenselauC: Preparing to read the ubiquitin code: characterization of ubiquitin trimers by top-down mass spectrometry. *J Mass Spectrom*.2016;51(4) : 10.1002/jms.3759 315-21 10.1002/jms.3759 27041663PMC4909354

# Peer review: Referee response for version 1

**DOI:** 10.5256/f1000research.11496.r20226

**Published:** 2017-02-21

**Authors:** John J Bergeron

**Affiliations:** 1 Department of Medicine, McGill University Hospital Research Institute, Montreal, Quebec, H3A 2B2, Canada

The author has provided a description of the methodological issues that were resolved by the author for a 2010 paper using hydrogen deuterium exchange and mass spectrometry to characterize the beta adrenergic GPCR. The article may be a response to another article published by another group in 2015 also on the use of hydrogen deuterium exchange and mass spectrometry to characterize the beta adrenergic GPCR. A recently published review ^1^ attempts to summarize how hydrogen deuterium exchange and mass spectrometry has transformed not only GPCR characterizations (the author of the submitted opinion article here, Dr. Zhang  is credited for his work in the review ^1^) but also the study of peripheral membrane proteins. In the Opinion article submitted by the author, Dr. Zhang, the two-step approach for the characterization of the beta-adrenergic receptor is indicated in section 1 and Fig1. Detergent considerations are indicated in sections 2,3 as well as Fig2. Proteinase digestion is considered in section 4, monovalent cation considerations in section 5, buffer concentrations in section 6 and automation in section 7. Three supplementary figures are used to compare sequence coverage using different protocols and an extensive 7 page supplementary section on detergent ( solubilisation) choices.

 It is difficult for this reviewer to see the conceptual advance in this submitted Opinion piece and that of the recent review by the same author ( Dr. Zhang) in a 2015 review in Molecular and Cellular Proteomics ( indeed some of the figures are similar for GPCR coverage and credited as such in this submitted Opinion piece ).

 One innovation that may be an extension of hydrogen deuterium exchange mass spectrometry may be the application of “Native” mass spectrometry ^2^ to integral membrane proteins such as the beta-adrenergic receptor.

 Perhaps the author could consider the important biological discoveries made through hydrogen deuterium exchange mass spectrometry for integral membrane proteins using, as an example, the beta adrenergic receptor and the hope that this can be extended though “Native” mass spectrometry, especially for resolving the dynamics of the interactions with the intracellular subunits of the hetero trimeric signaling complex.

I have read this submission. I believe that I have an appropriate level of expertise to confirm that it is of an acceptable scientific standard, however I have significant reservations, as outlined above.

## References

[1] VadasOJenkinsMLDornanGLBurkeJE: Using Hydrogen-Deuterium Exchange Mass Spectrometry to Examine Protein-Membrane Interactions. *Methods Enzymol*.2017;583: 10.1016/bs.mie.2016.09.008 143-172 10.1016/bs.mie.2016.09.008 28063489

[2] LeneyACHeckAJ: Native Mass Spectrometry: What is in the Name?. *J Am Soc Mass Spectrom*.2017;28(1) : 10.1007/s13361-016-1545-3 5-13 10.1007/s13361-016-1545-3 27909974PMC5174146
